# Feasibility, acceptability, and preliminary efficacy of yoga to improve maternal mental health and immune function during the COVID-19 crisis (Yoga-M_2_ trial): a pilot randomized controlled trial

**DOI:** 10.3389/fnhum.2023.1115699

**Published:** 2023-05-02

**Authors:** Rahul Shidhaye, Vidyadhar Bangal, Hemant Bhargav, Swanand Tilekar, Chitra Thanage, Suryabhan Gore, Akshada Doifode, Unnati Thete, Kalpesh Game, Vaishali Hake, Rahul Kunkulol

**Affiliations:** ^1^Department of Psychiatry, Pravara Institute of Medical Sciences, Loni, India; ^2^Department of Health, Ethics, and Society, Care and Public Health Research Institute, Faculty of Health, Medicine and Life Sciences, Maastricht University, Maastricht, Netherlands; ^3^Department of Obstetrics and Gynecology, Pravara Institute of Medical Sciences, Loni, India; ^4^Department of Integrative Medicine, National Institute of Mental Health and Neurosciences, Bengaluru, India; ^5^School of Public Health and Social Medicine, Pravara Institute of Medical Sciences, Loni, India; ^6^Directorate of Research, Pravara Institute of Medical Sciences, Loni, India; ^7^Department of Pharmacology, Pravara Institute of Medical Sciences, Loni, India

**Keywords:** yoga, mental health, immune function, rural, India, pregnancy

## Abstract

**Introduction:**

Women are vulnerable during pregnancy as they experience multiple physical and psychological problems which can lead to stress and poor quality of life ultimately affecting the development of the fetus and their health during and after pregnancy. Prior evidence suggests that prenatal yoga can improve maternal health and well-being and can have a beneficial effect on immune system functioning. To date, no study has been conducted in a rural, low-resource setting in India to assess the feasibility, acceptability, and preliminary efficacy of a yoga-based intervention on perceived stress, quality of life, pro-inflammatory biomarkers, and symptoms of upper respiratory tract infections.

**Methods:**

To address this gap and assess whether a yoga-based intervention could improve maternal mental health and immunity during the COVID-19 crisis (Yoga-M2 trial), a single-blind individual randomized parallel group-controlled pilot trial with a 1:1 allocation ratio was implemented. We randomly allocated 51 adult pregnant women, with gestational age between 12-24 weeks in the Yoga-M2 arm (*n* = 25) or the enhanced usual care arm (EUC) (*n* = 26). Feasibility and acceptability were assessed using the process data and In-Depth Interviews (IDIs) with the trial participants and yoga instructors. Multiple linear regression was used to compare follow-up scores for quantitative outcomes.

**Results:**

A three-month follow-up assessment was completed for 48 out of 51 participants (94.12%). We did not find any statistically significant difference between both arms in total Perceived Stress Scale scores, quality of life (Eq-5D-5L index), and serum C Reactive Protein levels at the three-month follow-up assessment. The critical barriers to practicing yoga were lack of knowledge about the benefits of yoga, lack of ‘felt need' to practice yoga, lack of time to practice, lack of space, lack of transport, and lack of peer group to practice yoga. Despite this, women who regularly practiced yoga described the benefits and factors which motivated them to practice regularly.

**Discussion:**

The learnings from this trial will help design the explanatory trial in the future and the study findings can also be used by the primary health care system to deliver yoga-based interventions in the newly created health and wellness centers.

**Trial registration:**

This trial was prospectively registered with the Clinical Trials Registry of India on 25 January 2022. https://www.ctri.nic.in/Clinicaltrials/showallp.php?mid1=65173&EncHid=&userName=CTRI/2022/01/039701. Trial registration number: CTRI/2022/01/039701.

## Introduction

Women experience several physical and psychological health problems during pregnancy. In a study from Sri Lanka, a very large proportion of women (90.3%) reported ill health during pregnancy leading to disturbances in their daily activities (Agampodi et al., 2013). Minor physical ailments during pregnancy have a huge impact on quality of life and wellbeing, which is also affected by stress, anxiety, and depression during the antenatal period (Dunkel Schetter and Tanner, 2012). This can lead to adverse maternal and fetal outcomes including pre-term birth, low birth weight, and depression after pregnancy and can also negatively affect neuro-cognitive development in infants and children (Gelaye et al., 2016). Prenatal yoga can play an important role in improving the health and wellbeing of “would-be” mothers. A recent systematic review and meta-analysis found beneficial effects of yoga on all important psychological outcomes: stress, anxiety, and depression as well as the quality of life (Corrigan et al., 2022). Yoga was also found to be safe during pregnancy (Corrigan et al., 2022).

The current study was designed against the backdrop of the Coronavirus disease (COVID-19) pandemic which severely disrupted human life across the globe during the years 2020, 2021, and most of 2022. COVID-19 affected the access and quality of health services delivered to non-COVID patients, especially the vulnerable sections of society such as pregnant women (WHO, 2020). Yoga was believed to be a potential intervention that could not only reduce stress and improve the wellbeing of pregnant women during the pandemic but could also improve immune system functioning and reduce the chances of respiratory tract infection (Falkenberg et al., 2018). Three Indian studies have assessed the effect of yoga on “perceived stress” during pregnancy (Satyapriya et al., 2009; Deshpande et al., 2013; Bhartia et al., 2019) and four other studies have assessed the effect of yoga on “physiological stress” (Field et al., 2013; Bershadsky et al., 2014; Hayase and Shimada, 2018; Kundarti et al., 2020). However, no study has combined the assessment of “perceived stress” with an inflammatory biomarker (Serum C-Reactive Protein), quality of life assessment, and assessment of symptoms of upper respiratory tract infection. Further, most of these studies were conducted with participants from urban areas with higher socio-economic backgrounds (Corrigan et al., 2022), and no study has been conducted in a rural low-resource setting in India. Previous studies have reported several barriers for women to participate in yoga sessions (Battle et al., 2019; Kinser et al., 2021).

A pilot randomized controlled trial was designed to address this knowledge gap with the aim of assessing the feasibility, acceptability, and preliminary efficacy of a yoga-based intervention to improve maternal mental health and immunity (Yoga-M_2_).

### Study objectives

1. To estimate participant eligibility, recruitment, retention-in-care, and study completion rates.
2. To assess the feasibility of delivering Yoga-M_2_ and the acceptability of this intervention by the participants.
3. To assess the preliminary efficacy of Yoga-M_2_ to improve maternal mental health and immune function.

The detailed trial protocol was published earlier (Shidhaye et al., 2022). In this study, we have presented the findings related to the study objectives and discussed the learnings from this pilot trial. This will help the design of a future explanatory trial to assess the effectiveness (and cost-effectiveness) of Yoga-M_2_ on stress, wellbeing, and immune system functioning during pregnancy among a rural population.

## Methods

### Trial design

Single-blind individual randomized parallel group-controlled pilot trial with a 1:1 allocation ratio.

### Important changes to methods after pilot trial commencement

No major changes were made in the methodology as described in the protocol paper after the commencement of the trial except that we also enrolled pregnant women from neighboring villages other than the four villages earlier described in the published protocol (Shidhaye et al., 2022). These villages were within a radius of 10 km of the Pravara Institute of Medical Sciences (PIMS). This was done as we had to complete the enrollment in 10 weeks. A few adaptations in the Yoga-M_2_ intervention were done and they are described below. In the case of one of the outcome measures [European Quality of Life-5 Dimensions (EQ-5D-5L)], we had proposed to use the value set/preference weights for Thailand to calculate a single utility score (Eq-index) as the Eq-5D value set for India was under development. However, the Indian value set was recently published (Jyani et al., 2022), and we used the same in our analysis.

### Participants

#### Eligibility criteria for participants

Pregnant women attending the antenatal clinic of the Department of Obstetrics and Gynecology, PIMS were contacted by a trained Research Assistant (RA). Those who expressed an interest in participating were screened by the RA using an eligibility assessment form. An inclusion/exclusion checklist was completed by the RA, and then, an obstetrician examined the participants to determine whether they could be enrolled in the study.

The following inclusion/exclusion criteria were used:

#### Inclusion criteria

- Adult pregnant women above 18 years of age
- Gestational age between 12 and 24 weeks
- Planning to stay in the study area throughout the study duration (~4 months)

#### Exclusion criteria

- Pregnant women who were advised by the obstetrician to take rest due to medical/obstetric problems
- History of two or more spontaneous abortions/miscarriages
- Inability to communicate in the Marathi language
- Inability to attend yoga sessions
- Receiving treatment for depression or any other mental health condition
- Practicing yoga regularly for at least one time a week for the past 4 weeks

### Settings and locations where the data were collected

The study was carried out in PIMS in Rahata taluka of Ahmednagar district in Maharashtra, India. The details of the study setting are provided in the study protocol paper (Shidhaye et al., 2022).

### How participants were identified and consented

Participants who fulfilled all the inclusion-exclusion criteria and were deemed appropriate to be enrolled in the trial by the obstetrician were provided the participant information sheet, and study procedures were explained to them. They were given sufficient time to read the information sheet and their concerns/queries about participation in the trial were addressed by the RA. If they agreed to participate, an informed written consent form was obtained. A copy of the participant information sheet and the informed consent form was handed over to the participants. To measure the level of serum C-Reactive Protein, 5 ml of blood was collected at baseline and at the 3-month follow-up assessment. The consent form included a specific item for the collection of the blood sample. Participants were also requested to provide consent to share the anonymized data with other researchers and to use the anonymized data to support other research in the future. A separate informed written consent form was completed at the time of baseline, the 3-month follow-up assessment, and when the participant was invited to participate in an in-depth interview as part of the qualitative evaluation. The informed consent procedure was completed in a separate room in the antenatal clinic to ensure privacy and confidentiality. The written informed consent was obtained as per the approved protocol (Shidhaye et al., 2022).

### Interventions

Participants in the intervention arm practiced Yoga-M_2_, i.e., yoga-based intervention for maternal mental health and immunity while those in the comparison arm received enhanced usual care (EUC).

### Yoga-based intervention for maternal mental health and immunity (Yoga-M_2_)

Yoga-M_2_ is an adaptation of the Integrated Approach for Yoga Therapy during pregnancy (IAYT-P) (Satyapriya et al., 2013). Given the rural context of the intervention delivery and the need to include activities to improve immune function, a few changes were made in the IAYT-P yoga sequence. Neck and shoulder movements were added for all the participants and one *asana* (“*viparit karani*”) was dropped for women who could attend yoga sessions only wearing a *saree* (a traditional Indian attire for women draped around the body and over one shoulder, and at times covering the head). The IAYT-P was usually delivered in a hospital setting, but in this trial, we decided to deliver the yoga sessions in a community hall or an *Anganwadi* center (rural mother and childcare center) closer to participants” homes. The details about the *Anganwadi* center are in the protocol paper (Shidhaye et al., 2022). We also included advice on diet, sleep hygiene, and physical activity for all participants. All these changes were made prior to the commencement of the trial and are described in the protocol paper along with the modified yoga sequence (Shidhaye et al., 2022).

The adaptions in Yoga-M_2_ after the commencement of the pilot trial are described below:

#### Frequency and intensity of intervention delivery

A 3-day training session to teach the entire yoga sequence to participants was completed in the beginning, and this was followed by weekly in-person group yoga sessions. Earlier, the plan was to have a 5-day training session, but the yoga instructors felt that the Yoga-M_2_ sequence could be taught in 3 days. Participants faced multiple difficulties in attending in-person yoga sessions, and they also suggested that it will be good to complete the initial training in 3 consecutive days. At the beginning of each yoga session, a safety checklist was completed by each participant.

After the initial training, participants were requested to continue the daily practice of yoga at home and attend one in-person supervised yoga session at least one time in 2 weeks (for the next 11 weeks).

#### Location of intervention delivery

Most of the yoga sessions were conducted in an *Anganwadi* center as per the original plan. A few sessions were conducted in PIMS and at participants” homes depending on the convenience of the participants and the yoga instructors.

#### Yoga sequence and session duration

No modifications were made to the yoga sequence other than those described earlier. Muslim participants were given the option to recite (or skip) the opening and closing prayer, fold hands in *namaskar* mudra, and chant *Omkar*. Yoga sessions were typically between 45 and 75 min.

#### Home practice

Participants were provided a booklet with details of all yoga activities and photographs at the time of randomization. They were encouraged by yoga instructors and the Intervention Coordinator (IC) to regularly practice yoga at home using the booklet. After the initial training session, instructors spent time individually with each participant to help them develop their home practice schedule. Specific changes/modifications for each participant were suggested based on the time of gestation and their individual health needs. Participants were briefed on how to maintain a log of their home practice and to maintain a record of injuries due to yoga practice.

#### Yoga instructors

Two women yoga instructors facilitated all in-person yoga sessions.

#### Health advice

During the initial training sessions, the yoga instructors discussed information related to sleep hygiene and diet during pregnancy. Educational leaflets containing this information were provided to the participants.

### Comparison arm: enhanced usual care (EUC)

A single health education session was delivered by the IC immediately after randomization to all participants in the EUC arm. This session included advice related to diet during pregnancy, sleep hygiene, and the benefits of regular physical activity during pregnancy. Similar to participants in the Yoga-M_2_ arm, they were also provided a summary of this advice in the form of an educational leaflet. Participants with a Perceived Stress Scale (PSS) score of > 13 were requested to visit a lady psychiatrist in the Department of Psychiatry, PIMS.

## Outcomes

### Feasibility and acceptability

1. Number and proportion of eligible participants recruited per week in each arm.
2. Number and proportion of recruited participants in the Yoga-M_2_ arm who attend the initial three in-person training yoga sessions.
3. Number and proportion of recruited participants in the Yoga-M_2_ arm who attend at least 50% of the six follow-up yoga sessions.
4. Number and proportion of recruited participants who completed the follow-up assessment 3 months post-randomization in each arm.

Participant satisfaction was assessed using a structured feedback form. In-depth interviews (IDI) with participants were undertaken to understand the benefits of participation in the trial, barriers to practicing yoga, and overall feedback on intervention delivery-related aspects such as yoga sequence booklet, educational leaflets, and logs to be completed at home. IDIs with both yoga instructors were done to specifically understand the barriers to delivering the yoga-based intervention to pregnant women.

### Preliminary efficacy

Validated Marathi language versions of PSS, EuroQoL 5 Dimensions Score (EQ-5D-5L), Wisconsin Upper Respiratory Symptom Severity Scale (WURSS), and estimation of serum C-Reactive Protein levels were used to assess the preliminary efficacy. The rationale for using these outcome measures and the details of the assessments are described below. No changes were made in the assessments or measurements after the pilot trial commenced, and they are similar to what is described in the protocol paper (Shidhaye et al., 2022).

#### Perceived stress scale (PSS)

Perceived stress was measured using the 10-item version of the original scale (Cohen et al., 1983). The items in this instrument assess how unpredictable, uncontrollable, and overloaded participants appraise the situations in their lives in the recent month. PSS has been widely used in research studies across the globe and it has acceptable psychometric properties (Lee, 2012). In this study, we used the Marathi version of the PSS, which was translated from the English version using the World Health Organization (WHO) guidelines for the process of translation and adaption of instruments (WHO, 2013).

#### EuroQoL 5 dimensions score (EQ-5D-5L)

European Quality of Life Five Dimension (EQ-5D-5L) descriptive system was used to measure the health-related quality of life (HRQoL). A single utility score was calculated using the recently published value set/preference weights for India (Jyani et al., 2022). Participants also provided an overall evaluation of their health using a visual analog scale (VAS). The validated Marathi version of the EQ-5D-5L was used with permission from the EuroQoL Group (Herdman et al., 2011). The EQ-5D has been validated in different parts of the world (Feng et al., 2021).

#### Wisconsin upper respiratory symptom severity scale (WURSS)

The incidence and severity of upper respiratory tract infections in the participants were assessed using the Wisconsin Upper Respiratory Symptom Survey (WURSS-21). This is a 21-item scale where 10 items assess symptoms, nine items assess functional impairments, and one item each assesses the overall severity and change. Responsiveness, reliability, convergence, and importance to patients on this scale have been validated (Barrett et al., 2005). Participants completed the WURSS-21 at the end of each day of the study. We used the Marathi version of WURSS with permission from the developers.

#### Serum C-reactive protein (CRP)

Serum CRP was included as an outcome measure to assess the effect of yoga on the inflammatory process. CRP is a non-specific marker of the inflammatory process, and the serum levels increase with body insult, tissue damage, aging, and cardiovascular diseases. Bacterial (or viral, fungal) infections lead to a marked increase in serum CRP levels. Although it is not a diagnostic marker, it can be used to track the inflammatory process, overall wellness, and the individual”s quality of life. Lifestyle changes such as cessation of smoking, increasing physical activity, and reducing body mass index lead to a decrease in serum CRP levels (Kao et al., 2006). Yoga and other mind-body techniques reduce inflammation through a reduction in CRP (Morgan et al., 2014). Approximately 5 ml of venous blood sample was collected from each trial participant at the baseline and 3-month follow-up assessment. Serum CRP levels were determined using the fixed-point immune-rate method (VITROS 250/350/5, 1 FS/4600/XT 3400 Chemistry Systems, and the VITROS 5600/XT 7600 Integrated Systems). The limit of detection for VITROS Chemistry Products CRP slides is 2.72 mg/L.

### Timing of the outcome assessment

PSS, EQ-5D-5L, and WURSS were administered at the baseline (just before randomization) and the follow-up (3 months post-randomization).

A log to document injuries/non-serious adverse events during the duration of the trial was maintained by participants in both arms.

### Sample size

The sample size calculation for the pilot trial was based on the recommendations by Whitehead et al. (2016). Assuming the standardized effect size between the two arms of 0.2, we recruited 25 pregnant women in each arm (total = 50).

### Randomization

The baseline assessment of all the participants was completed immediately after they submitted the informed written consent form. A structured interview schedule which included data on demographic and socio-economic measures and the primary and secondary outcome measures was used by the RA to complete the baseline assessment.

### Sequence generation

The randomization list was generated using the statistical program R (version 3.6.1) by an independent statistician. Variable block sizes (randomly selected block sizes of 2/4/6/8) were created followed by a list of intervention allocations (Yoga-M_2_ or EUC) using a 1:1 allocation ratio. The list was (only) shared with the Clinical Research Coordinator (CRC) based in the Directorate of Research, PIMS. He prepared serially numbered, opaque, sealed envelopes using the randomization list.

### Allocation concealment mechanism

Participants were randomly allocated to the intervention group by the IC using serially numbered, opaque sealed envelopes after they completed their baseline assessment.

### Implementation

After the baseline assessment of the participants was completed, treatment allocation was undertaken by the IC in a separate room. The RA who completed the baseline assessment was not aware of the treatment allocation. During the trial recruitment phase, every morning the IC collected a set of opaque sealed envelopes from the CRC as per the serial numbers. The IC opened the opaque sealed envelopes (as per the serial numbers) in front of the participants who had completed the baseline assessment. They were informed about the intervention group (i.e., Yoga-M_2_ or EUC) and were requested to not reveal their group assignment to the RA at the 3-month follow-up assessment. To maintain allocation concealment, neither the PI, the RA, nor any staff working at the hospital had access to the randomization lists. The IC completed the randomization form, placed it in the envelope, and returned all the open envelopes to the CRC at the end of the day. Based on the returned envelopes, the CRC maintained the record of intervention allocation.

### Blinding

Due to the nature of the intervention, participants, their family members, and study team members involved with intervention delivery (IC and yoga instructors) were aware of the participants” assigned intervention during the trial. A baseline and three-month follow-up assessment were completed by the RA who worked independently and did not interact with the intervention team members during the course of the trial. The obstetricians were also not aware of the intervention allocation status which minimized the risk of any change in their behavior while providing routine antenatal services to the participants.

We monitored the risk of contamination at the level of the yoga instructors, intervention coordinator, and participants. Yoga instructors and the intervention coordinator were requested to not share information about Yoga-M_2_ with pregnant women other than those enrolled in the intervention arm (Yoga-M_2_).

### Statistical methods

#### Quantitative analysis

Descriptive statistics (means and standard deviations or proportions) were used to present trial feasibility outcomes and to compare the demographic and socio-economic characteristics of trial participants at the baseline. For preliminary efficacy outcomes, the primary analysis was intention-to-treat. Multiple linear regression was used to estimate regression coefficients and the 95% confidence intervals for continuous outcomes. Analyses of continuous outcomes additionally adjusted for the baseline values of that outcome. For the quality of life (EQ-5D-5L) outcome, the EQ-5D index was calculated using the recently published Indian value data set. The EQ-5D index has a value attached to a particular EQ-5D health profile based on people”s preferences about their state of health. Statistical analyses were conducted in R (version 3.6.1). The STATA code to estimate EQ-5D-5L is available online. This code was appropriately modified and was used in R.

IDIs were audio-recorded, and field notes were taken. Interviews were conducted in Marathi. During data collection, debriefing meetings were held, and feedback was provided to the interviewers (IC and an additional Research Assistant not involved in any other trial procedures). IDIs were first transcribed and then translated into English. Bilingual team members, fluent in English and Marathi carried out regular back-translation checks. The first author, IC, and other members of the research team read and re-read the English transcripts to develop a codebook for coding the data. The first author then coded all the transcripts and additional codes were inductively added from the data. Once the data was coded, it was summarized, and key themes were extracted based on discussions with the research team members. NVivo 9 software (QSR) was used for qualitative data analysis.

A Trial Steering Committee (TSC) provided oversight for the trial. It comprised four members who were all independent of the study team. All members were women and one of them was a participant representative (she was pregnant during the duration of the trial). The TSC met four times, once before the beginning of the trial, twice during the implementation of the trial, and finally after the enrollment ended. The TSC was regularly updated about the trial progress and the trial findings were shared with them before the submission of the manuscript.

#### Role of funding agency

The funding agency did not play any role in the design, implementation, evaluation, and analysis of the trial findings.

## Results

### Participant flow

**Figure 1:**
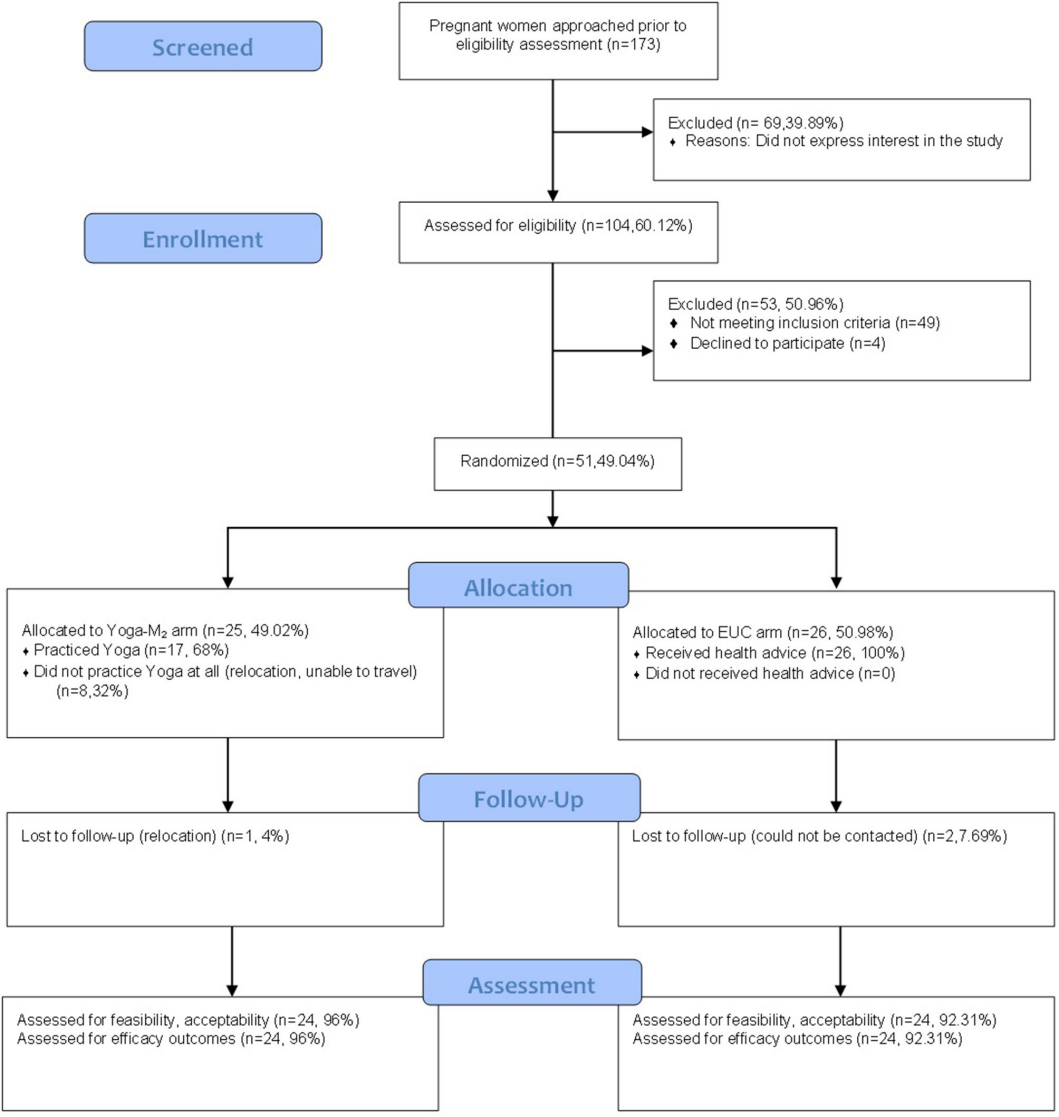
Participant flow chart.

We contacted 173 pregnant women in the antenatal clinic of the Department of Obstetrics and Gynecology, PIMS. Out of these 173 pregnant women, 104 of them (60.12%) expressed interest in the study and were assessed for eligibility. Almost half of the participants (*n* = 49,47.12%) did not meet the inclusion criteria and a few declined to participate (*n* = 4, 3.85%). The rest of the participants (*n* = 51, 49.04%) were enrolled in the study. See Figure 1 for the participant flow chart with reasons for non-inclusion.

### Recruitment

Participants were enrolled over 9 weeks (03 March 2022 to 05 May 2022) and the average number of participants enrolled per week was 5.67. Of the 6 working days in a week, we were able to enroll participants only on two (or at the most three) days per week due to the limited availability of the obstetrician.

A 3-month follow-up assessment was completed for 48 out of 51 participants (94.12%). One participant went to her mother”s place (left the study area), while two other participants could not be contacted over the telephone. Additionally, we were unable to collect blood samples for 11 other participants at the 3-month follow-up assessment as they were unable to come to the hospital due to transportation issues or lack of time. Their follow-up assessment was completed at home (*n* = 7) or over the phone (*n* = 4).

### Baseline data

The baseline characteristics of trial participants are presented in Table 1. There was no statistically significant difference in any of the demographic or socio-economic characteristics of trial participants in both arms.

**Table 1:** Comparison of demographic and socio-economic characteristics of trial participants at baseline (*n* = 51).

**Variable**	**Intervention group (Yoga-M_2_) (*n* = 25) *n* (Row %)**	**Control group (Enhanced usual care) (*n* = 26) *n* (Row %)**	***p*-value**
**Age (in years)**			
≤ 20	9 (50.00)	9 (50.00)	0.864
21-25	13 (52.00)	12 (48.00)	
≥26	3 (37.50)	5 (62.50)	
**Age (in years)** (Mean ± SD)	22.12 ± 3.50	22.88 ± 4.08	0.476
**Gestational age (in weeks)** (Mean ± SD)	18.08 ± 4.32	17.58 ± 3.38	0.646
**Education**			
Secondary School (Grade 10) or below	16 (45.71)	19 (54.29)	0.555
Higher Secondary School (Grade 11) or above	9 (56.25)	7 (43.75)	
**Religion**			
Non-Hindu (Muslim)	5 (71.43)	2 (28.57)	0.248
Hindu	20 (45.45)	24 (54.55)	
**Caste**			
Scheduled Castes/Tribes	10 (50.00)	10 (50.00)	0.910
General	15 (48.39)	16 (51.61)	
**Occupation**			
Housewife	21 (47.73)	23 (52.27)	0.847
Agricultural workers	2 (50.00)	2 (50.00)	
Professional	2 (66.67)	1 (33.33)	
**Type of house**			
*Kaccha* (temporary structure of bamboo, grass, thatch etc.)	8 (53.33)	7 (46.67)	0.764
*Pucca* (permanent structure of stone, reinforced cement concrete)	17 (47.22)	19 (52.78)	
**Type of family**			
Joint	20 (46.51)	23 (53.49)	0.465
Nuclear	5 (62.50)	3 (37.50)	
**Land ownership**			
Yes	16 (53.33)	14 (46.67)	0.572
No	9 (42.86)	12 (57.14)	
**Below poverty line**			
Yes	8 (40.00)	12 (60.00)	0.392
No	17 (54.84)	14 (45.16)	
**Type of pregnancy**			
Primigravida	13 (44.83)	16 (55.17)	0.686
Multigravida	12 (54.55)	10 (45.45)	
**Perceived stress scale score** (Mean ± SD)	9.44 ± 6.66	10.81 ± 6.17	0.451
**EQ-5D-5L (EQIndex)** (Mean ± SD)	0.95 ± 0.08	0.94 ± 0.08	0.673
**Serum CRP** (Mean ± SD)	7.10 ± 4.73	9.50 ± 6.80	0.168

### Numbers analyzed

For the outcome related to the feasibility of yoga sessions, the total number of participants in the Yoga-M_2_ arm was 25. For preliminary efficacy outcomes, the total number of participants in both arms was 48 (24 in each arm).

### Outcomes and estimation

#### Feasibility and acceptability of yoga-based intervention (Yoga-M_2_) delivery

Out of the total 25 participants enrolled in the Yoga-M_2_ arm, 12 (48%) attended three consecutive in-person yoga training sessions. Four participants (16%) attended one session each while one participant (4%) attended two sessions. Eight participants (32%) did not attend any yoga sessions.

Six participants attended at least 50% of the possible six follow-up in-person supervised yoga sessions.

The yoga sessions were mostly conducted in the morning, and 46 out of 70 (65.71%) sessions were conducted from 10.30 AM to 12.30 PM. This was the time slot most suitable for the participants as they could complete their household work before 9.30 AM and could then attend the session with at least half an hour interval between their light meal in the morning and the yoga session. Another more acceptable time slot was 3.00 to 4.00 PM, 11.43% of sessions happened during this time slot.

Home practice logs were completed by nine (75%) participants out of the 12 participants who regularly attended supervised yoga sessions. The mean duration of daily home practice was 39 min (SD: 14.09), and it ranged from 15 to 61 min. The median duration was 40 min with an interquartile range of 35–47 min.

### Participant satisfaction

A structured satisfaction survey was completed by 10 participants at the end of the 3-day in-person training session. Half of them (*n* = 5) rated their overall experience as “very good”, and the rest of them rated it as “good. ” Activities included in the yoga sequence were rated “very good” by 70% of participants and “good” by 30% of participants. Except for one participant, others rated “style of instruction,” “opportunity to ask questions to instructor,” and “discussion with her' as “very good” or “good.” One participant was not satisfied and rated these items as “can”t say”. The duration of the yoga sessions was rated “very good” by 40% of participants and “good” by the remaining 60% of participants. Overall, there was high satisfaction with the yoga sessions, and they used the following words to describe their experience, “inspirational” (*n* = 5), “health-promoting” (*n* = 4), “pleasant,” “simple,” and “useful,” (*n* = 3). Other phrases such as “novel,” “stimulating,” “challenging,” as well as “boring,” and “useless” were also used one time.

### Preliminary efficacy

The findings for all the outcomes below are summarized in Table 2.

**Table 2:** Summary of the findings related to the preliminary efficacy outcomes.

**Outcome variable**	**Group**	**Baseline**	**3-month follow-up**	**Differences with 95% Confidence Interval and** ***p*****-value**	
				**Within-group**	**Between-group** ^*^
PSS (Mean ± SD)	Yoga-M_2_	9.44 ± 6.66 *n* = 25	8.37 ± 5.52 *n* = 24	−0.62 (−2.18,3.43) 0.649	0.93 (−2.07,3.95) 0.537
	EUC	10.81 ± 6.17 *n* = 26	7.96 ± 5.12 *n* = 24	−2.96 (−0.04,5.96) 0.053	
EQ-5D-5L (EQIndex) (Mean ± SD)	Yoga-M_2_	0.95 ± 0.08 *n* = 25	0.94 ± 0.10 *n* = 24	0.03 (−0.01,0.07) 0.122	−0.01 (−0.06–0.04) 0.629
	EUC	0.94 ± 0.08 *n* = 26	0.93 ± 0.08 *n* = 24	0.01 (−0.03,0.04) 0.759	
Serum CRP (Mean ± SD)	Yoga-M_2_	7.10 ± 4.73 *n* = 21	9.38 ± 10.33 *n* = 17	−0.74 (−2.89,1.41) 0.468	−1.52 (−6.03,2.99) 0.496
	EUC	9.50 ± 6.80 *n* = 24	9.24 ± 6.51 *n* = 20	−0.81 (−5.52,3.91) 0.724	

PSS, Perceived Stress Scale, EQ-5D-5L, European Quality of Life, 5 Dimensions, 5 Levels, Serum CRP=Serum C-Reactive Protein.

^*^Comparison between the two arms at 3-month follow-up adjusted for baseline values.

### Perceived Stress Scale (PSS)

At the 3-month follow-up assessment, the adjusted mean difference in the PSS score between the two arms was 0.93 (95% CI: −2.09–3.95). This difference was not statistically significant.

The mean PSS score in the Yoga-M_2_ arm at the 3-month follow-up was 8.37 (SD = 5.52). The mean difference of 0.62 (SD = 6.64) between the two time points was not statistically significant (*p* = 0.649). The mean PSS score in the EUC group at the 3-month follow-up was 7.96 (SD = 5.12). The mean difference of 2.96 (SD = 7.11) between the two time points was not statistically significant (*p* = 0.053).

The variation in difference was 2.33 (favoring the EUC group), and the 95% confidence interval for this difference was −6.33–1.66, thus not statistically significant.

The median change (decrease) in the PSS score in the EUC group was 0.5 and the interquartile range was −1 to 3.25. There was an extreme reduction in PSS score in four participants, and these can be termed as outlier observations. Any observation with a change in PSS scores beyond (or below) 1.5 times the interquartile range of the third quartile (or first quartile), i.e., the upper or lower fence was termed as an outlier. This definition of an outlier is as per the National Institute of Standards and Technology, the United States Department of Commerce (https://www.itl.nist.gov/div898/handbook/prc/section1/prc16.htm). In the Yoga-M_2_ group, the median decrease in PSS score was 1 (inter-quartile range: −3.25–5). There were no outlier observations in the Yoga-M_2_ group.

If we removed the four outlier observations, the difference in the difference between the two groups was 0.48 (favoring the Yoga-M_2_ group). This difference was also not statistically significant (95% CI: −2.70–3.65).

### EuroQoL 5 dimensions score (EQ-5D-5L)

The difference in EQindex between the two groups at the 3-month follow-up assessment was not statistically significant (adjusted mean difference = −0.01, 95% CI: −0.06–0.04).

In the Yoga-M_2_ group, the mean EQindex at the three-month follow-up was 0.934 (SD = 0.099). The mean difference of 0.029 (SD = 0.122) between the two time points was not statistically significant (*p* = 0.58). The mean EQindex in the EUC group at the 3-month follow-up was 0.933 (SD = 0.076). The mean difference of 0.005 (SD = 0.081) between the two time points was not statistically significant (*p* = 0.759).

The variation in the difference was 0.024 (favoring the EUC group), and the 95% confidence interval for this difference was −0.025–0.073, thus not statistically significant.

### Wisconsin upper respiratory symptom severity scale (WURSS)

The completed WURSS log was submitted by 32 (62.74%) participants, 13 in the Yoga-M_2_ arm and 19 in the EUC arm. Symptoms of upper respiratory tract illness were reported by 8 out of 13 (61.54%) Yoga-M_2_ participants and 13 out of 19 (68.42%) EUC participants. Symptoms were present for an average of 11.44 days (SD: 11.23) in Yoga-M_2_ participants and 23.85 days (SD: 15.48) in EUC participants.

### Serum C-reactive protein (CRP)

In the Yoga-M_2_ group, the mean serum CRP at the 3-month follow-up was 9.38 (SD = 10.33) and the median serum CRP level was 5. The mean serum CRP level in the EUC group at the 3-month follow-up was 9.24 (SD = 6.51) and the median serum CRP level was 6.2.

The adjusted mean difference in serum CRP levels between the two groups at their 3-month follow-up assessment was −1.52 (95%CI: −6.03–2.99), thus not statistically significant.

### Qualitative study findings

We completed 17 IDIs with trial participants, of which 13 were from the Yoga-M_2_ arm and four were from the EUC arm. Almost all participants (10 out of 12) who attended three in-person yoga training sessions were interviewed. The rest of the two participants had attended one session and one participant was unable to attend any session. IDIs with both yoga instructors were also completed.

The findings of the IDIs are presented in Table 3. Based on the study objectives we identified the following themes: (a) benefits of yoga practice (and health advice), (b) overall feedback on intervention delivery, (c) barriers to practicing yoga, and d) facilitators for yoga practice. The first two themes are related to the acceptability of the intervention (Yoga-M_2_ and the EUC), and the rest are concerned regarding the feasibility of the intervention. A summary of the findings is presented below.

**Table 3:** Qualitative findings based on in-depth interviews.

**Theme**	**Sub-theme**	**Indicative quotes**
**Objective: Acceptability of the intervention**		
**Benefits of yoga practice**	Physical benefits Participants mentioned that they felt *refreshed* after yoga practice and the body felt *lighter*. Reduction in body ache, muscle pain, swelling over legs, nausea, and vomiting. A few participants said, ‘*anga mokala hota'* (in Marathi) which loosely translates to sense of loosening/relaxation in English.	*I do housework, I mean I was not doing it before, I felt tired, but after doing yoga I got strength to do housework*. (Trial participant, Yoga-M2 arm, IDI) *My abdominal muscle pain and swelling got better, leg swelling reduced, vomiting and nausea also reduced*. (Trial participant, Yoga-M2 arm, IDI)
*Feeling calm after doing yoga. Madam said to take a deep breath and let it out, sit quietly for a while and sleep will come automatically*. (Trial participant, Yoga-M2 arm, IDI)
		*I was not able to sleep before yoga, now after yoga I sleep better*. (Trial participant, Yoga-M2 arm, IDI)
**Benefits of health advice (both arms)**	Changes in sleep, diet, physical activity Two participants in the EUC group regularly read the educational leaflet and made changes in their sleep, diet, and physical activity, while other two participants in the EUC group categorically said that they did not make any changes in their sleep, diet, or physical activity. In the Yoga-M2 group, nine out of 12 participants made changes in their daily routine based on the information in the educational leaflet.	*I eat two to three times a day. After eating, I do not sleep for two hours. First, I was eating heavy food. Now I eat green leafy vegetables and fruits. I used to sleep in the daytime, but now I don't sleep the whole day. Now I sleep on time at night, take eight hours of sleep, and get complete rest. After getting that booklet and after following it I felt a lot of change in my own body*. (Trial participant, EUC arm, IDI) *We changed our routine as we read the book. I stopped watching mobile and watching TV, so now my sleep is complete, and I feel fresh*. (Trial participant, EUC arm, IDI)
**Intervention delivery** **(Yoga-M2 arm)**	Yoga instructor Participants felt comfortable in the presence of the instructor.	*She explained every exercise well and did it with me. In case of difficulty, she explained to me how to do it*. (Trial participant, Yoga-M2 arm, IDI)
	Various yoga related activities and other health advice was delivered well. Yoga sessions Participants preferred group yoga sessions as they enjoyed interacting with other participants. A few of them created a whatsapp group for internal communication and helped each other in completing the home practice logs. Home practice logs If there were any difficulties in completing the home practice logs, they took the help from yoga instructors or their peers. None of the participants who were interviewed said that it was boring to complete the logs.	*She taught yogasanas well. She explained the benefits of each yoga asana and told us to relax if there is any problem*. (Trial participant, Yoga-M2 arm, IDI) *Liked in the group. I don't like to practice yoga alone*. (Trial participant, Yoga-M2 arm, IDI) *It was a bit difficult to understand but my friends* (other trial participants) *were there. We contacted each other and told each other and filled the books*. (Trial participant, Yoga-M2 arm, IDI)
**Enhanced Usual Care arm**	Satisfaction with intervention allocation Some participants in the EUC arm contacted yoga instructor and expressed their desire to practice yoga.	*Well, some women who went to control group even requested me personally, ma'am we want to do yoga, because we are in control group, we don't get to do yoga. We are really lucky that we got opportunity to participate in this study but now we are in control group, so what should we do?* (Yoga instructor, IDI)
**Objective: Feasibility of the intervention**		
**Barriers to practice yoga**	Misconceptions about yoga Some participants (and their family members) were afraid and had misconceptions about yoga practice. Yoga was perceived to be harmful during pregnancy. Yoga instructors also identified such misconception as an important barrier.	*Now the group we had is from rural areas. They had only heard the concept of 'Yoga' and seen it somewhere. They think that yoga is not for us, or whether it is necessary for us and there were many doubts in their minds about yoga. The biggest challenge was to gain their trust. There was disbelief because their previous generation or someone in the family had never done anything like this. They knew that common people could do yoga, but they found it a little difficult to believe that such a thing should be done during pregnancy……*. *There were some superstitions in their mind related to yoga. The family members did not understand. There were some problems in the family……It means not to go out of the house, or we were not allowed to come after a certain month as something may happen if someone's shadow falls on pregnant woman*. (Yoga instructor, IDI)
	Attitude toward yoga According to yoga instructors some participants felt that pregnancy was a *routine* thing and that no additional efforts were needed to be healthy. Yoga instructors highlighted this lack of felt need and mentioned that participants did not think that yoga practice was ‘for them'. Yoga was seen as an additional ‘*work*' imposed by yoga instructors.	*During the sessions, I realized that they do yoga as a work as we have to do the work in the house. This pregnancy is also a work, and it has to be done*. (Yoga instructor, IDI)
	Minor ailments during pregnancy	*Sometimes their health is not good, so every day during pregnancy there can be these changes taking place. Sometimes there is mood change, sometimes mental state is not good, sometimes physical state is not good then in this situation they can't practice yoga*. (Yoga instructor, IDI)
	Lack of time and space Participants were quite occupied with household chores and were unable to find free time during the day to practice yoga. Half of the participants had previous children and had to spend good amount of time taking care of them. Festivals and guests at home were also cited as barriers. Most of the participants lived in a two-room house with at least three other family members. This provided very little space for them to practice yoga at home.	
	Transportation Participants were dependent on their husband (or other family member) to bring them to yoga center.	*Yes, there were problems when my husband went to work, there was no one to drop me for a yoga session. He had to come from work and drop me. After that he had to reach back after that session to drop me at home… After it was over, that madam called me again and said if you can do extras, come home, but I couldn't so she used to come to my house to take my yoga session*. (Trial participant, Yoga-M2 arm, IDI)
**Facilitators for yoga practice**	Benefits of yoga practice	*It doesn't feel right. Today I avoided it (yoga). If I would have done yoga, I would not have suffered*. (Trial participant, Yoga-M2 arm, IDI)
	Perceived benefit on the growth and development of fetus	*I practiced yoga for my health and for the health of my baby*. (Trial participant, Yoga-M2 arm, IDI)
	Advice from the obstetrician	*I was told by the doctor that if you do yoga, you will be better and if you don't you will become weak, and this pain will continue*.
	Family support Father-in-law of one of the participants was a regular yoga practitioner. He along with his wife encouraged one participant to practice yoga at home. Interestingly, two daughters of this participant also joined her in yoga practice.	*It was good along with my daughters and father-in-law too*. (Trial participant, Yoga-M2 arm, IDI)
	One participant discussed with her sister who in-turn advised her about the benefits of yoga. This resulted in regular yoga practice by this participant.	*I have support from my husband and sister. I asked her about yoga. How is yoga, should we do it. So, she told that, her neighbor also practiced yoga. So, I thought that I should do it*. (Trial participant, Yoga-M2 arm, IDI).
	One participant felt very bored being alone at home. Yoga practice reduced boredom of this participant and she got interested in regular practice of yoga.	
	Regular follow-up by the research team	*No, I thought something. They come* (home) *or call, and it is for our benefit. They come a lot of times, there is some benefit (*for me)*, they come because of us*. (Trial participant, Yoga-M2 arm, IDI)
		*Their phone call made me remember that we must do something, they care and so we should also be taking care of ourselves*. (Trial participant, Yoga-M2 arm, IDI)

All participants (except one) in the Yoga-M_2_ group reported physical and/or psychological benefits due to yoga practice. Participants in the EUC group also reported benefits related to the advice they received immediately after randomization. A single session of health education included information related to sleep hygiene, changes in diet during pregnancy, and regular physical activity, and an educational leaflet summarizing this advice was given to all participants (including those in the Yoga-M_2_ group). All participants reported that they had a good experience interacting with yoga instructors. Almost all participants preferred the group format for yoga training. Participants regularly referred to the yoga manual and found the photos and instructions very useful. However, most of the participants in the EUC arm (three out of the four interviewed) were not satisfied with the random allocation of the intervention. They wanted to practice yoga during pregnancy. One participant mentioned that she watched YouTube videos and practiced yoga.

Several barriers to yoga practice were reported by the trial participants and the yoga instructors involved in the delivery of the intervention. These can be broadly categorized as lack of knowledge regarding the benefits of yoga, lack of “felt need” to practice yoga, lack of time to practice, lack of space, lack of transport, and lack of peer group to practice yoga. The first two factors operate at the level of an individual participant, the next two are family and household-related issues, and the last two are related to the format of intervention delivery. Yoga is part of the school curriculum (in the study area) and many participants practiced yoga during school days. However, this was discontinued after they left the school. At the time of pregnancy, most participants were unaware that yoga practice could be beneficial during pregnancy. Participants were motivated to attend yoga sessions as well as continued practice of yoga at home due to several factors. One of the key factors was the practice of yoga itself. Many participants experienced the benefits of yoga practice. This motivated them to continue the practice on regular basis. Advice from the doctor, support from the family members, and positive interaction with the yoga instructor were other important factors that motivated participants.

### Safety

The injury log was submitted by 31 (60.78%) participants, of which 13 were in the Yoga-M_2_ arm and 18 in the EUC arm. In the Yoga-M_2_ arm, body aches and muscular pain were reported by 8 (61.54%) participants and the mean duration was 6.8 days (SD: 7.83). The median rating of ache/pain was 4.25 (on a scale of 1 to 10, the latter being most severe). Only one participant in the Yoga-M_2_ arm attributed the pain to yoga practice. In the EUC arm, body aches, and muscular pain were reported by 9 (50%) participants, the mean duration was 6.78 days (SD: 3.83). The median rating of ache/pain was 4.00.

### Serious adverse event

One participant in the Yoga-M_2_ arm had a stillbirth. She had attended only two yoga sessions in the first 2 weeks after randomization. Twelve weeks later, she underwent a caesarian section for fetal distress. She had a fresh stillbirth. At the time of the caesarian section, the participant was COVID-positive. This serious adverse event was not considered to be related to the intervention. The Principal Investigator reported this to the Institutional Ethics Committee and Clinical Research Coordinator submitted an independent report to the Trial Steering Committee.

### Trial implementation costs

The total cost incurred for the completion of the trial was INR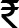 133,299 (USD$ 1,625.6 in nominal terms at an exchange rate of 82 INR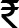 per USD$, and USD$ 6165.5 in purchasing power parity terms at 21.6 INR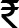 per USD$). Two-thirds (67.1%) of the funds were spent on intervention delivery which included yoga instructor honorarium for conducting in-person yoga sessions (INR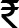 52,000), travel allowance for participants (INR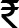 7,500), and costs for printing yoga manual (INR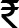 29,999). The rest of the expenses (32.9%) were related to research evaluation: serum CRP estimation (INR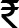 35,000) and remuneration to all participants for the assessments (INR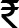 8,800).

## Discussion

In this study, we described the findings of a pilot randomized controlled trial of yoga-based intervention to improve maternal mental health and immunity (Yoga-M_2_) intervention. This was probably the first trial of a yoga-based intervention to improve the maternal wellbeing of women from a rural low-resource setting in India. It was feasible to recruit pregnant women in a short duration of 9 weeks and retain almost all of them till the end of the trial. There were several barriers in organizing yoga sessions (described below); however, 48% of the participants attended three in-person yoga training sessions and half of them attended at least 3 follow-up in-person yoga sessions, thus a total of six sessions. Yoga-M_2_ was acceptable to pregnant women as they described the benefits of yoga practice on their health and factors which motivated them to continue practicing yoga after the initial training sessions. We found no statistically significant difference between both arms in total PSS scores, quality of life (Eq-5D-5L index scores), and serum CRP levels at the 3-month follow-up assessment.

It was feasible to enroll the proposed number of participants in a very short duration (9 weeks), despite the ongoing (although receding) COVID-19 pandemic in the study area. The majority of the pregnant women whom we contacted expressed interest in participating in the study (60.15%) and only very few (3.85%) declined to participate even though they fulfilled all the inclusion criteria. Pregnant women were predominantly contacted in the antenatal clinic of a tertiary hospital. The clinic caters to ~200 pregnant women every day. These women mostly come in the morning after traveling a long distance and are quite concerned about the long queues and waiting time. This was the prominent reason for pregnant women attending the antenatal clinic not agreeing to the eligibility assessment.

The retention rate in the trial was 94.12% and except for three participants, all completed follow-up assessments. Of these, 81.25% of assessments were completed within a 7-day window after the due date (of follow-up assessment) as proposed in the protocol paper (Shidhaye et al., 2022), 10.42% within 14 days after the due date, and 8.33% of assessments were done between 14 and 21 days after the due date. The retention rate in other yoga-based intervention studies for the mental health and wellbeing of pregnant women ranged from 65% to 92% (Sheffield and Woods-Giscombé, 2016). Blood sample collection for serum CRP did pose feasibility problems. The process was time-consuming as pregnant women had to go to the central lab in the institute for providing the blood sample. For most of the participants, we combined serum CRP assessment with their routine antenatal blood investigations. However, serum CRP was not assessed for a few participants (*n* = 5) at baseline as the lab personnel missed the instructions to assess the same. Follow-up assessments were planned with the routine antenatal visit of the participants, but this was not possible always. In the case of 11 participants, follow-up visits had to be conducted at home as they were unable to come to the hospital within the assessment window or had to be done by telephone as the participants had relocated to their mother”s home for delivery. Daily completion of the WURSS checklist for 90 consecutive days tended to be an onerous task in the beginning; however, 62.74% (32 out of 51) of participants submitted the same at the time of follow-up assessment. Approximately a similar proportion of participants (58.82%, 30 out of 50) submitted the injury logs. The rest of the participants had either misplaced the logs or had forgotten to bring them along at the time of the follow-up assessment. None of the participants whom we interviewed said that it was burdensome to maintain the logs.

Organizing yoga sessions for participants in the intervention arm was quite challenging. We did anticipate transportation as an important challenge earlier and to mitigate the same yoga sessions were conducted in an *Anganwadi* center closer to the participant”s home. An *Anganwadi* caters to a population of ~1,000 individuals, and in rural areas, it indicates that the distance between two consecutive *Anganwadis* is short enough for pregnant women to walk. Due to the centralized enrollment in a tertiary hospital, it was almost impossible to enroll multiple pregnant women from the same Anganwadi (or the neighboring one), in the same intervention arm, and around the same period. Thus, enrolled participants in the Yoga-M_2_ arm were living far off from each other and were also at different stages of the study enrollment making it extremely difficult to organize large group yoga sessions for the participants. It was possible to organize small group sessions (three to four participants) at a time and for quite a few of them (*n* = 7), individual one-on-one sessions were organized in an *Anganwadi* closer to their home or at their home. This barrier, in our opinion, is quite unique to rural areas which are sparsely populated.

Almost all yoga sessions were organized during the months of March, April, and May when summer is at its peak in India. This is also the marriage season in India and schools have holidays. These factors limited the availability of yoga instructors as well as that of participants, reducing the number of follow-up yoga sessions. The obstetrician was also available for a limited number of days during the week and was extremely busy with the ongoing clinical work, which effectively reduced the time available for enrollment and participant interaction.

In India, women prefer to live with their parents during the time of delivery. Residence in the study area during the entire period of study (12 weeks) was one of the inclusion criteria, and all the participants enrolled did mention that they were not planning to relocate. However, the decision was not always entirely taken by women. Two participants went to their mother's place 1 week after they were enrolled in the study, and although they were very keen to practice yoga, they could not attend even a single session. Two participants wanted to come for yoga sessions, but there was an accident in the family in the case of one of them and there was no one at home to bring her to the session for the other participant. Hence, they could not attend any yoga sessions.

Participants who attended the first three in-person yoga sessions (training sessions) as well as follow-up sessions expressed their satisfaction with the way sessions were conducted, the manner the yoga instructors reported the benefits of yoga practice, and also mentioned factors that motivated them to undertake regular yoga practice. In the IDIs, participants who were randomized to the Yoga-M_2_ arm mentioned that it was good to get into the yoga group while three out of four participants in the EUC arm clearly expressed dissatisfaction about not being allocated the yoga arm. Only two participants in the Yoga-M_2_ group declined to attend yoga sessions citing health reasons. The above findings indicate the overall acceptability of the intervention amongst the study group. Interestingly, participants in the EUC arm were happy to receive a single session of health education, read and followed the educational leaflets provided to them, and made certain changes in their daily routines. They also reported the benefits of advice pertaining to sleep, diet, and physical activity.

The Perceived Stress Scale (PSS) has been used as an outcome measure by four RCTs; three from India (Satyapriya et al., 2009; Deshpande et al., 2013; Bhartia et al., 2019) and one from Japan (Hayase and Shimada, 2018). A meta-analysis that included these studies reported a standardized mean difference of 1.03 (95% CI: 0.52–1.55) between the yoga and the control group (Corrigan et al., 2022). In all three Indian studies, perceived stress significantly reduced in the yoga group between the baseline and the follow-up while it increased in the control group. In our study, perceived stress was reduced in both arms, and the reduction was higher in the EUC arm, although none of these differences were statistically significant. There are two possible explanations: lower baseline PSS scores in our study (in both arms) and the effect of health advice on participants in the EUC arm. The mean baseline PSS score in a study by Bhartia et al. was 19.25 (SD: 2.10) in the yoga arm and in another study by Satyapriya et al. was 15.9 (SD:5.01) in the yoga arm. Their final follow-up assessment scores were also higher than our baseline scores. We had referred participants in the EUC arm with PSS>13 to psychiatrists in PIMS. One participant in the EUC arm who had a very high PSS score at baseline consulted a psychiatrist and she was also engaged in group antenatal care activities (outside the trial). She said that she immensely benefitted from these activities and her follow-up PSS score was 2. This also happened with a few other EUC participants who followed the health advice provided as part of the single session, and they had a huge reduction in PSS score at follow-up. We did not find any study from India which has used EQ-5D-5L to assess the health-related quality of life during pregnancy. Health-related quality of life peaks during the early second trimester followed by a decrease, and it is lowest in the late third trimester (Wu et al., 2021). This study also found a slight decrease in health-related quality of life measured using EQindex. It is important to note that participants in this study had a better quality of life in the early second trimester (mean EQindex = 0.95, SD = 0.08) compared to that reported in the above-mentioned study from China (mean EQindex = 0.89, SD = 0.12). This may partly explain the lack of any change in the quality of life of our trial participants.

In their systematic review and meta-analysis, Corrigan et al. have highlighted several methodological issues in the yoga trials implemented in pregnant women. The strength of our pilot RCT is that it attempted to address many of these issues. First, the randomization sequence was generated by a statistician who was completely independent and not involved in any of the study procedures. Baseline assessments were completed prior to randomization and the RA who conducted them was not aware of intervention allocation during any part of the study. Thus, single-blinding was effectively achieved and this being a type of behavioral intervention, double-blinding at the participant level was anyway not possible. Both arms were balanced at baseline and the loss to follow-up was similar (and very minimal) in both arms minimizing the risk of selection bias. As per the suggestions by Corrigan et al., frequency, intensity, duration, and type of yoga-based intervention have been described in this study. All the tools used for outcome assessments were validated and only one trained RA assessed all participants by reducing the risk of information bias.

There are three important limitations of this study. First, the sample size does not provide adequate power to assess the efficacy of the intervention. This was inevitable though, as the study was designed as a pilot trial to understand the feasibility of delivering a yoga-based intervention. The funding for this trial was limited and only for 1 year. Second, our sample population was predominantly from a lower educational background (68.63% of women were educated up to secondary school/grade 10 or below). In our previous study from the same setting, which looked at the demand for yoga among pregnant women, 49.10% of women had completed secondary school (or below) (Shidhaye et al., 2021). This limits the generalizability of the findings to rural women with less education, and it is likely that the barriers we noticed may not exactly be the same for a different sample with higher educational status. The third limitation was our inability to assess the fidelity of intervention delivery. Although we had discussed this in the study protocol and provided the fidelity assessment checklist, we could not video record the yoga sessions which could be rated by an independent assessor.

The key learnings from this pilot trial are in the following domains: (a) pre-enrollment activities, (b) enrollment and retention, (c) assessments, and (d) intervention content and delivery. Due to the ongoing COVID-19 pandemic and limited resources (research team members, funding, and time), we did not have the opportunity to engage with local communities. Pregnant women were contacted directly in a busy antenatal center in a tertiary care setting and a good proportion of them (39.85%) did not complete the eligibility assessment as they were in hurry. Our first key lesson is that this challenge can be mitigated by community engagement activities such as organizing small-group meetings in a local setting (e.g., an *Anganwadi* center) to explain to women the nature of the study and to explore if they would be interested in participating. We also realized that it is difficult for women to commit to participating in this kind of study as the opinion (as well as consent/permission) of other family members is important. Community meetings will provide us an opportunity to invite the family members of pregnant women and explain the study. Women (and their in-laws), although generally aware of yoga, were not very clear if yoga could be safely practiced during pregnancy. This underlines the importance of conducting awareness sessions about the potential benefits (and safety) of practicing yoga during pregnancy, which can help pregnant women (and their family members) to make an informed decision about study participation.

The second important lesson is regarding stronger adherence to the inclusion criteria of “residence in the study area.” Relocation of enrolled women to their maternal home will pose a serious barrier in a future trial, but this can be addressed by having more open and multiple discussions with potential participants (and their family members). Instead of an antenatal center, enrollments can be planned in a community setting, making the process more feasible. An obstetrician (or a trained medical officer) can assess women either in the community or remotely using digital platforms.

Third, all the assessments, especially those which require blood sample collection should be done in a health facility/primary health center closer to the participant's home, which could eliminate the need to travel to a central antenatal care unit in a tertiary center (as in our case) and will help to improve the feasibility of trial assessments. We could also plan to collect home logs for injury and WURSS assessment from participants on a weekly/fortnightly basis ensuring greater compliance with these assessments. Participants in both arms reported improvements in their sleep. In this trial, we did not assess the sleep quality which needs to be added in a future trial.

Finally, the most important lesson is regarding the delivery of Yoga-M_2_ intervention. It is not feasible (and sustainable) to conduct home-based (or *Anganwadi-*based) yoga sessions for a single participant. A group of women needs to be formed for the supervised yoga sessions. For this to happen, a greater number of women from a geographically contiguous area need to be enrolled in a given span of time enabling the formation of small groups. This will be possible with a community-based enrollment of participants. *Anganwadi* or a community center needs to be identified in advance for organizing yoga sessions. Once pregnant women are trained in the basic yoga sequence, follow-up sessions could be video-based and facilitated by a member of the project team. This may help in improving intervention adherence as a greater proportion of enrolled participants will be able to complete more yoga sessions. The intervention team will also have to intensely follow up with participants to encourage them to undertake the regular home practice of yoga and maintain the log of same.

## Conclusion

The findings of this pilot randomized controlled trial and the learning described above will inform the design of an explanatory trial which we plan to undertake in the near future. We found that it was feasible to organize yoga sessions in community settings, and the yoga-based intervention including the core yoga sequence used in this trial was acceptable to pregnant women. As part of this trial, a structured yoga manual was developed, which would serve useful in the next trial. We also strongly feel that our findings can be used by the primary health care system in Ahmednagar district particularly (and Maharashtra generally) to deliver yoga-based interventions in health and wellness centers created as part of the “Ayushman Bharat Yojana” (National Health Protection Scheme). We plan to widely disseminate the findings of this trial with key public health officials of the district and the state to ensure the same.

## Trial protocol

Trial protocol published earlier:

Shidhaye, R., Bangal, V., Bhargav, H., Tilekar, S., Thanage, C., Suradkar, R., et al. (2022). Yoga to improve maternal mental health and immune function during the COVID-19 crisis (Yoga-M 2 trial): study protocol for a pilot randomized controlled trial. [version 2; peer review: 2 approved]. *Wellcome Open Res*. 7, 109. doi: 10.12688/wellcomeopenres.17729.2.

## Data availability statement

The original contributions presented in the study are included in the article/Supplementary material, further inquiries can be directed to the corresponding author.

## Ethics statement

The studies involving human participants were reviewed and approved by Institutional Ethics Committee of the Pravara Institute of Medical Sciences (Approval Number: PIMS/DR/RMC/2020/225). The patients/participants provided their written informed consent to participate in this study.

## Author contributions

RS: conceptualization, methodology, validation, formal analysis, data curation, writing-original draft, visualization, supervision, and funding acquisition. VB: conceptualization, methodology, writing-review and editing, and supervision. HB: conceptualization, methodology, and writing-review and editing. ST: methodology, formal analysis, data curation, and writing-review and editing. CT, SG, AD, VH, and UT: investigation and writing-review and editing. KG: data curation and writing-review and editing. RK: writing-review and editing. All authors read and approved the final draft of the paper.
